# Cochlear Implantation of Bilaterally Deafened Patients with Tinnitus Induces Sustained Decrease of Tinnitus-Related Distress

**DOI:** 10.3389/fneur.2017.00158

**Published:** 2017-04-25

**Authors:** Steffen Knopke, Agnieszka J. Szczepek, Sophia Marie Häussler, Stefan Gräbel, Heidi Olze

**Affiliations:** ^1^Department of Otorhinolaryngology, Head and Neck Surgery, Charité – University Hospital Berlin, Campus Virchow-Klinikum, Berlin, Germany; ^2^Department of Otorhinolaryngology, Head and Neck Surgery, Charité – University Hospital Berlin, Campus Charité Mitte, Berlin, Germany

**Keywords:** cochlear implantation, hearing impairment, health-related quality of life, tinnitus-related distress, depressive symptoms, anxiety

## Abstract

**Objective:**

Tinnitus is a common symptom of hearing impairment. Patients who are bilaterally hard of hearing are often affected by tinnitus. However, they cannot undergo any of the standard tinnitus therapies, since they rely on hearing. Cochlear implantation (CI) used to treat severe hearing disabilities, such as bilateral hearing loss, was also shown to reduce tinnitus. Our goal was to determine if CI induces sustained reduction of tinnitus. We performed prospective, longitudinal analyses of tinnitus-related distress in a uniform group of bilaterally deafened patients after CI.

**Patients and Methods:**

The homogenous sample consisted of 41 patients who met the inclusion criteria and were consecutively included in this study. The impact of unilateral CI on tinnitus-related distress, health-related quality of life (HRQoL), and hearing abilities was studied with validated instruments. The follow-up appointments were scheduled at 6, 12, and 24 months after CI surgery. During the appointments, hearing abilities were estimated with monosyllabic Freiburg test, whereas the tinnitus-related distress, the HRQoL, and the subjective hearing were measured with standard questionnaires [Tinnitus Questionnaire (TQ), Nijmegen Cochlear Implantation Questionnaire, and Oldenburg Inventory, respectively].

**Results:**

Tinnitus-related distress decreased significantly from the mean TQ score of 35.0 (SD = 19.6) prior to surgery to the mean TQ = 27.54 (SD = 20.0) 6 months after surgery and remained sustained low until the end of follow-up period. In addition, CI significantly improved the hearing abilities and the HRQoL of all patients.

**Conclusion:**

The results from our prospective study suggest that in a homogenous sample of bilaterally deafened, implanted patients who report having tinnitus prior to surgery, CI alone not only improves the hearing abilities but also significantly reduces the tinnitus-related distress and improves the HRQoL in a sustained way.

## Introduction

Tinnitus is a common symptom of hearing impairment (1–3). Therapeutic use of hearing aids to treat mild-to moderate hearing loss was demonstrated to correlate with a decrease of tinnitus (4), although the data generated by clinical research neither support nor dismiss the use of hearing aids in tinnitus treatment (5). Of all types of hearing impairment, the most cumbersome is the severe bilateral hearing loss, which is often treated with cochlear implantation (CI) (6–10). Bilateral hearing impairment affects 12.7% (30 million) of the US Americans above 12 years of age, and the prevalence of bilateral hearing impairment increases with age (11). We and others have previously reported the incidence of tinnitus among the bilaterally hearing-impaired patients ranging between 70 and 90% (12–14) and making tinnitus a serious complaint in this particular group of patients.

Already decades ago, clinical observations linked the CI-mediated hearing recovery with the reduction in tinnitus (15–17). Ever since, various studies addressed the relationship between cochlear implants and tinnitus (12); however, the outcomes of the studies were somewhat conflicting. There are three main reasons for this: the first is varying sample size (from 1 to 26); the second is using different follow-up times (from 1 to 24 months) (18, 19); and the third is that despite recent recommendations to measure tinnitus-related distress before and after CI (10), the methods and the domains vary extremely from study to study (20). Furthermore, the design of clinical trials is often retrospective and the patients included have various types of hearing impairment (21–23). Moreover, the methods of treatment are frequently dissimilar and include unilateral hearing impairment treated with unilateral CI to bilateral hearing impairment treated with bilateral CI.

In our earlier studies, we concentrated on measuring the influence of CI on the quality of life (24), tinnitus-related distress, and psychological comorbidities (13, 14). We have demonstrated significant improvement of all domains measured following the CI. However, the follow-up time was rather short (13) and the patient sample was not homogenous (14).

The outstanding question in the field is how the cochlear implants affect tinnitus and tinnitus-related distress. The full answer to this question will be possible upon accumulation of high-quality evidence. This, in turn, can only be achieved by using specific batch of standardized validated instruments and by applying prospective longitudinal design to the studies.

Our present aim was to study tinnitus-related distress in a relatively homogenous group of patients over a longer period after CI. Our main question was if in this defined cohort, tinnitus-related distress improves solely upon auditory rehabilitation, and if yes, if this improvement is sustained over longer period.

## Patients and Methods

### Inclusion Criteria

The patients of both genders were consecutively included in the study upon signing written consent. Following inclusion criteria were used:

- diagnosis of bilateral severe or profound hearing loss with speech recognition ≤40% in the Freiburg Monosyllabic Test in quiet and with hearing aid; 65-dB sound pressure level
- tinnitus
- meeting of the clinical criteria for CI:
  ○ possibility to use general anesthesia
  ○ exclusion of retrocochlear disorder (e.g., vestibular schwannoma)
  ○ unremarkable cochlear anatomy
  ○ motivation for postoperative audiological rehabilitation
  ○ post-lingual deafness.

### Description of Study

**Figure 1:**
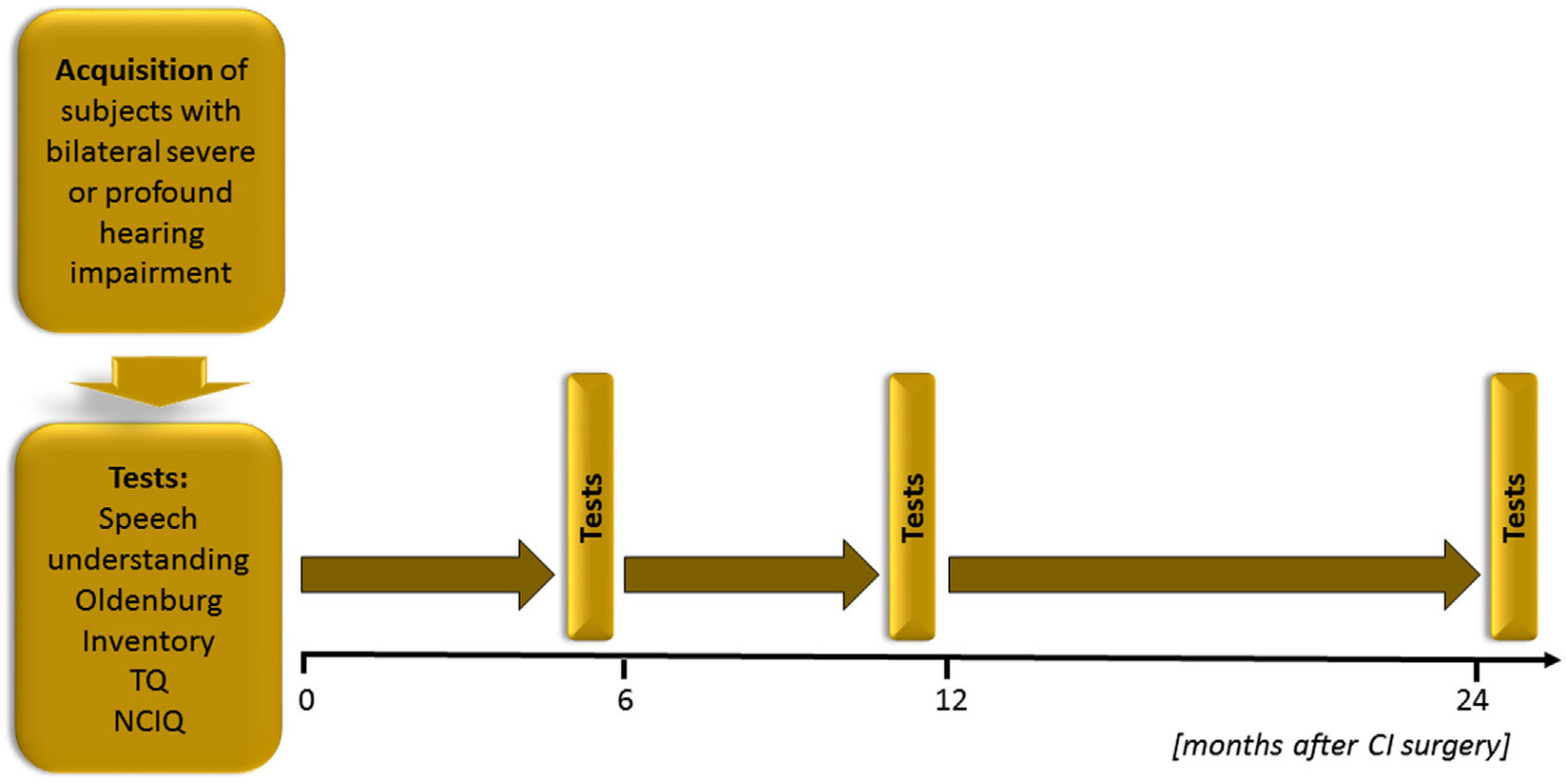
**Study design**.

Forty-one patients met the inclusion criteria and were followed for 2 years after CI. The data were collected between 2009 and 2016; the patients were admitted to the hospital between April 2009 and May 2014 for unilateral CI, and their last follow-up appointment was scheduled between July 2011 and February 2016. The appointments were scheduled at 6, 12, and 24 months after the surgery (see Figure 1). There were 22 women and 20 men in the sample—descriptive statistics are presented in Table 1.

**Table 1:** **General patients’ characteristics**.

	Mean	Minimum	Maximum	SD
Age	61	25	81	13.45
Duration of hearing impairment (years)	18.82	1	67	18.84
Percent of speech recognition using monosyllabic Freiburg test 65-dB sound pressure level on the ear scheduled for implantation	7.86	0	40	13.21

### Test Performed

All patients were audiologically examined. In addition, they were asked to complete psychometric questionnaires before surgery and during each consecutive appointment. The audiological tests and psychometric questionnaires used were previously described in detail (14, 25) and are presented in Table 2.

**Table 2:** **Questionnaires used in this study**.

Health-related quality of life (HRQoL): Nijmegen Cochlear Implantation Questionnaire (NCIQ)	NCIQ is a validated tool designed to determine the HRQoL of implanted patients. The three main domains “physical,” “psychological,” and “social” are derived from six subdomains: 1 Basic sound perception 2 Advanced sound perception 3 Speech production 4 Self-esteem 5 Activity 6 Social interactions. The score ranges from 0 (very bad) to 100 (optimal).
Speech perception: Freiburg Monosyllabic Test	The Freiburg Monosyllabic Test was used to determine the preoperative speech recognition in silence at 65-dB sound pressure level with optimized hearing aid and postoperative with cochlear implant again as well
Subjective audiological assessment: Oldenburg Inventory (OI)	Data were collected preoperatively and postoperatively about the subjective hearing with the OI. The OI additionally includes a total score in 3 categories: “hearing in quiet,” “hearing with background noise,” and “localization.” The 12 closed questions about everyday situations were marked with points from 1 to 5. The higher the score, the better the subjective hearing
Tinnitus distress: Tinnitus Questionnaire (TQ)	The tinnitus distress can be determined with TQ (26). Collected data represent 6 subdomains: emotional and cognitive distress, intrusiveness, auditory perceptual difficulties, sleeping disturbances, somatic complaints. The mean value is used to determine tinnitus grade: light (0–30 points), average (31–46 points), high (47–59 points), and very high (60–84 points). In addition, separation into compensated (≤46 points) and decompensated (47–84 points) tinnitus can be done based on the total score. The test–retest reliability is 0.94 for the total value and between 0.86 and 0.92 for the subscales. Cronbach’s α is 0.94 for the total value of the TQ and between 0.74 and 0.92 for the subscales

### Statistical Evaluation

For the statistical analyses, SPSS version 23 was used. Normal distribution was tested prior to statistical analysis using the Shapiro–Wilk test and a histogram. Because of lack of normal distribution in the majority of dataset, the Wilcoxon signed-rank test was used to compare the scores before and after CI. Correlations between Tinnitus Questionnaire (TQ) and Nijmegen Cochlear Implantation Questionnaire (NCIQ) scores were performed by computing the Spearman’s rank correlation coefficient.

## Results

### Postimplantation Sustained Decrease of Tinnitus-Related Distress

**Figure 2:**
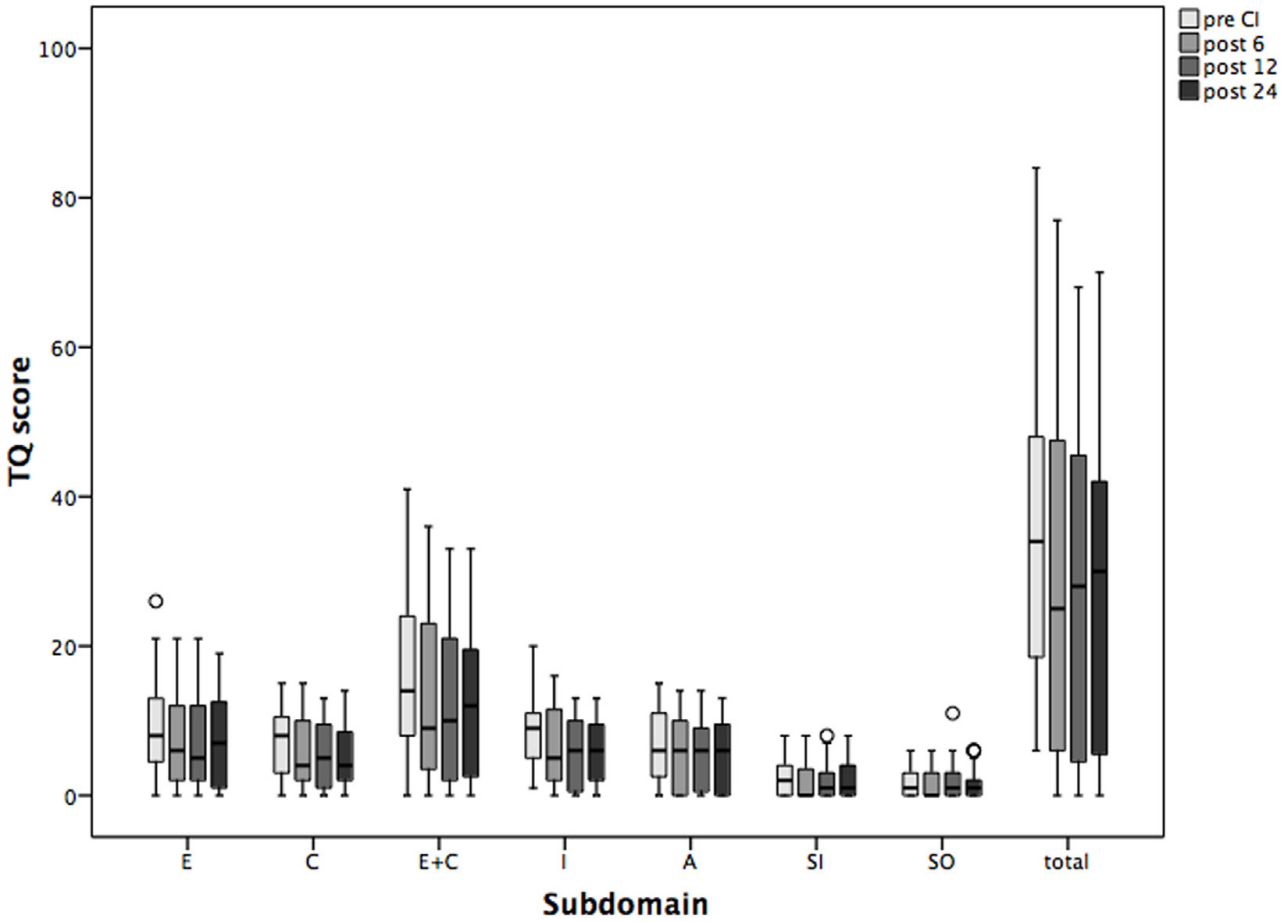
**Changes in tinnitus distress [Tinnitus Questionnaire (TQ)] and its subscales over the period of 2 years following cochlear implantation (CI)**. Shown are mean values and the range. Shown are mean values and the 95% CI. Pre CI, before CI; post 6, post 12, and post 24, 6, 12, and 24 months after surgery. E, emotional distress; C, cognitive distress; I, intrusiveness; A, auditory perceptual difficulties; SI, sleeping disturbances; SO, somatic complaints; total, total value.

Tinnitus was the main inclusion criterion. Prior to CI, the mean TQ score reflecting tinnitus-related distress was 35 (Table 3). TQ score decreased significantly already 6 months after CI, and this improvement was sustained over the 24-month follow-up period (Figure 2). Significant improvement of tinnitus-related distress was noted in 64.5% of all patients in the cohort. Regarding the individual TQ subscales, the emotional and cognitive distress were significantly reduced 12 and 24 months after implantation but the intrusiveness of tinnitus-related distress decreased already 6 months after surgery and stayed on a significantly lower level as compared to that before CI (Table 3). There was a trend in improvement regarding the subscales “auditory perception difficulties” and “somatic complaints,” but this trend has not reached the statistical significance.

**Table 3:** **Changes in parameters measured as compared to their values prior to cochlear implantation**.

	Prior to surgery			6 months after surgery			12 months after surgery			24 months after surgery		
	Mean	Median	SD	Mean	Median	SD	Mean	Median	SD	Mean	Median	SD
SR 65-dB sound pressure level (SPL)	7.9	0.0	13.2	40.4**	43.8	35.9	41.6**	35.0	34.2	43.2**	46.3	25.2
NCIQ 1	40.1	39.4	20.9	59.6**	60.0	20.0	62.1**	65.0	22.4	57.6**	60.0	18.1
NCIQ 2	42.4	40.0	19.2	56.1**	55.0	20.5	54.9**	55.0	21.0	53.2**	52.5	19.4
NCIQ 3	64.4	67.5	22.6	72.1	75.0	20.4	66.9	72.2	20.6	71.7	71.9	24.0
NCIQ 4	42.6	41.7	17.5	50.9**	45.0	19.7	52.9**	52.5	18.9	53.8**	50.0	21.4
NCIQ 5	37.9	37.5	18.2	49.1**	47.5	22.8	49.3**	47.5	23.0	47.5**	43.8	18.6
NCIQ 6	41.5	40.0	22.5	51.2**	50.0	25.7	53.0**	52.8	23.3	50.3**	50.0	18.9
NCIQ total	53.8	43.2	58.7	56.6**	55.1	18.5	57.5**	55.5	19.8	55.8**	56.8	15.2
TQ E	9.0	8.0	6.0	7.4	4.0	6.8	7.1*	5.0	6.6	7.2	7.0	6.4
TQ C	6.9	7.5	4.5	5.7	4.0	5.0	5.4*	4.5	4.4	5.6	4.5	4.5
TQ E + C	15.9	14.0	10.0	13.1	9.0	11.5	12.3*	9.5	10.9	12.7*	13.0	10.6
TQ I	8.2	9.0	4.1	6.1**	5.0	5.2	5.6**	5.5	4.7	5.8**	6.0	4.3
TQ A	6.6	6.0	4.8	5.2	5.0	4.9	5.1	5.5	4.7	5.2	5.5	4.5
TQ SI	2.5	2.0	2.4	1.8*	0.0	2.5	1.9	1.0	2.3	2.3	1.5	2.7
TQ SO	1.8	1.5	1.7	1.3	0.0	2.0	1.8	0.5	2.4	1.5	1.0	1.9
TQ total	35.0	33.5	19.6	27.5*	23.0	24.0	26.7**	26.5	22.8	27.6*	30.0	22.3
OI quiet	2.4	2.4	0.9	3.4**	3.6	1.0	3.4**	3.4	0.8	3.4**	3.4	0.9
OI noise	1.8	1.6	0.6	2.7**	2.6	1.0	2.7**	2.5	0.9	2.6**	2.4	0.8
OI localization	1.9	2.0	0.8	2.8**	3.0	1.0	2.7**	2.5	1.0	2.7**	2.5	1.0
OI total	2.1	1.9	0.7	3.0**	2.9	0.9	3.0**	2.9	0.8	2.9**	2.8	0.8

Asterisks indicate significant differences between respective variables when compared to their values prior to surgery as per Wilcoxon signed-rank test; *p ≤ 0.05, **p ≤ 0.01.

*SR, speech recognition (Freiburg Monosyllabic Test, 65-dB SPL); NCIQ, German version of Nijmegen Cochlear Implantation Questionnaire (1 basic sound perception; 2 advanced sound perception; 3 speech production; 4 self-esteem; 5 activity; and 6 social interactions); TQ, Tinnitus Questionnaire (E, emotional distress; C, cognitive distress; E + C, combined psychological distress; I, intrusiveness; A, auditory perception difficulties; SI, sleep disturbances; SO, somatic complaints; total, total score); OI, Oldenburg Inventory*.

**Figure 3:**
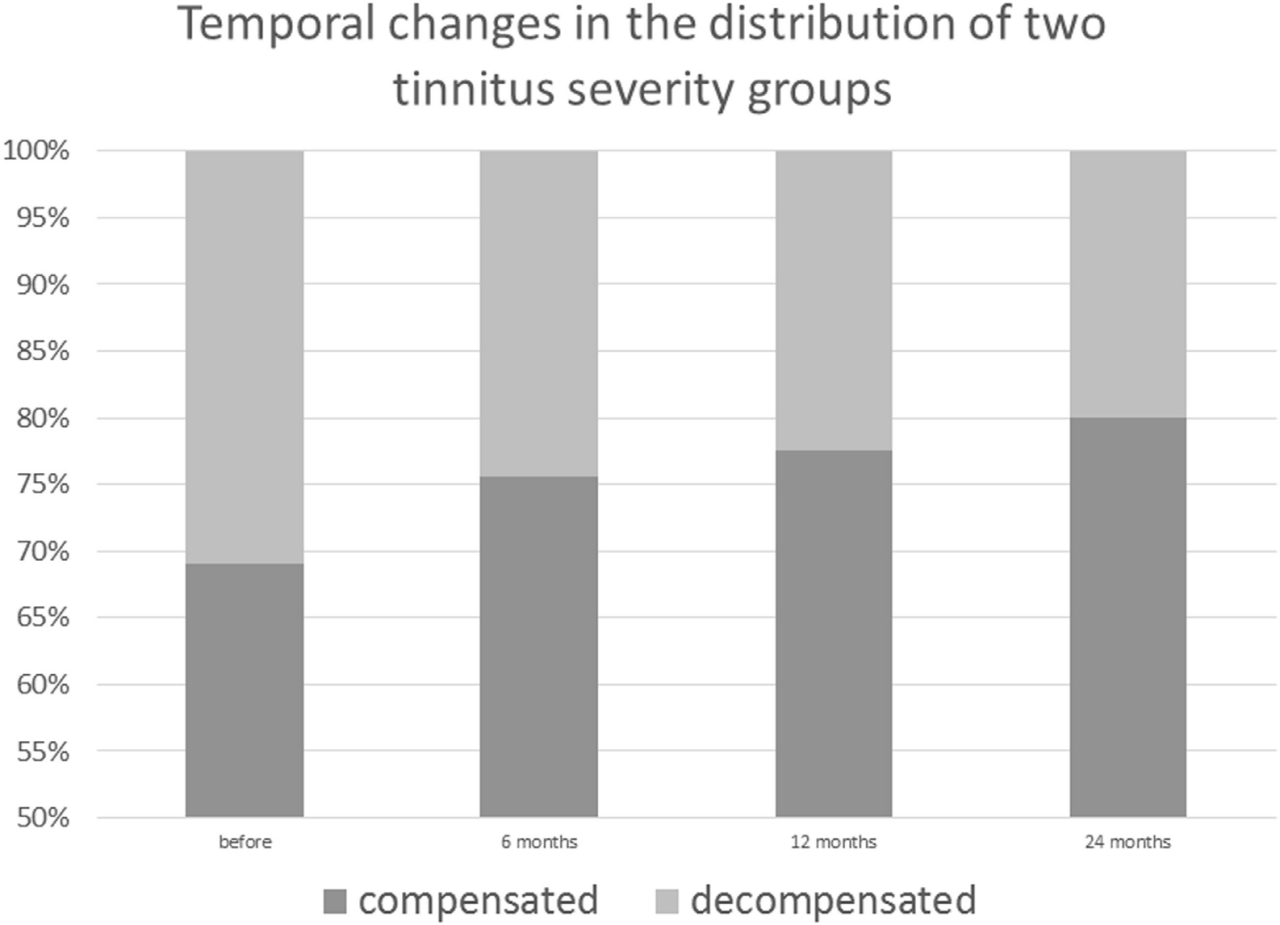
**Post-cochlear implantation decrease in decompensated, severe form of tinnitus among implanted patients**.

Prior to CI, 13 patients were affected by a severe, decompensated, tinnitus-related distress (TQ score = 47 or more). Six months after surgery, four patients had TQ scores on the compensated level, 12 months after surgery, five patients were compensated, and 24 months later, seven patients were compensated. In two patients with compensated TQ scores prior to surgery, tinnitus-related distress progressed further to the severe, decompensated form after CI (Figure 3).

### Post-Surgery Improvement of the Health-Related Quality of Life (HRQoL), Speech Perception, and Auditory Performance

**Figure 4:**
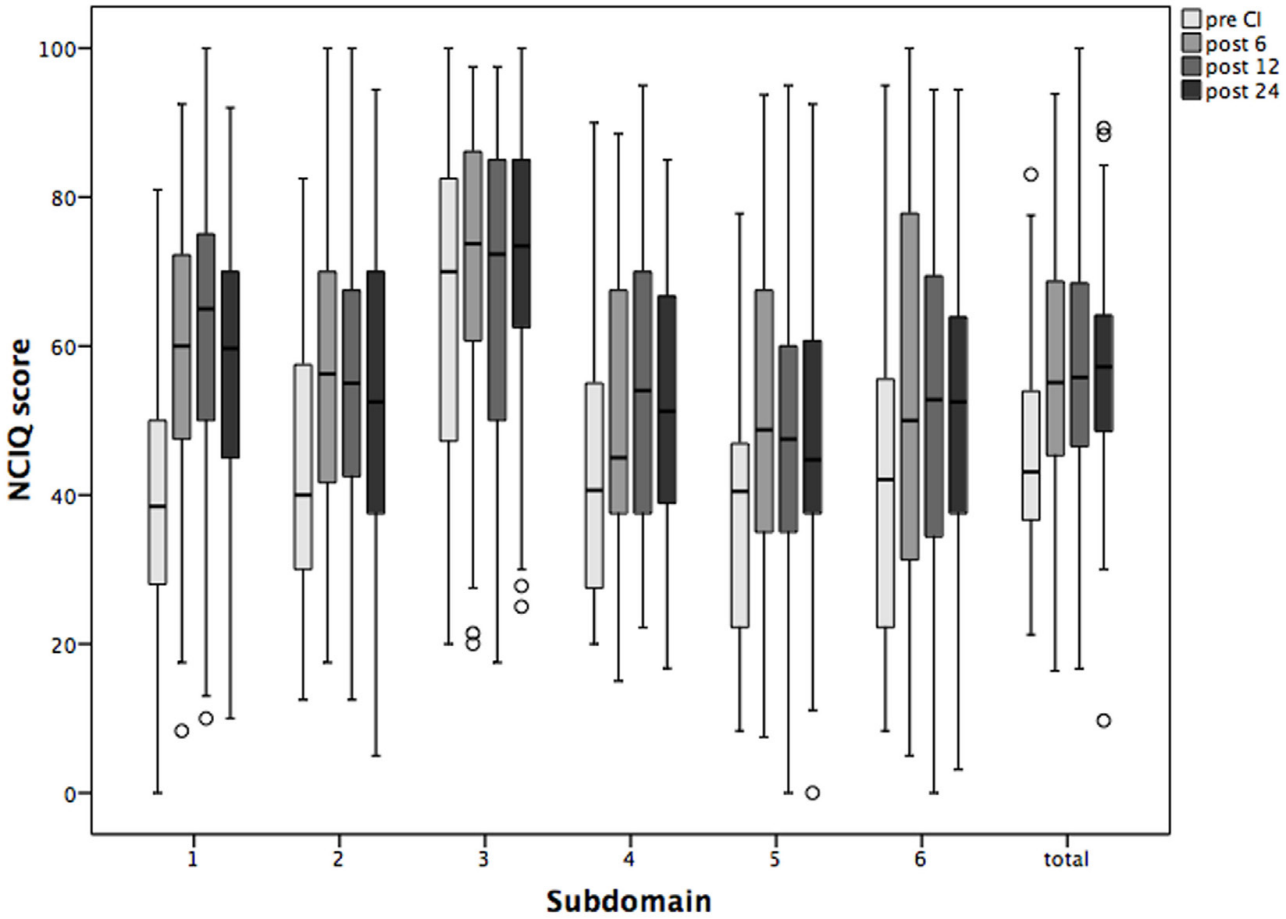
**Changes in the health-related quality of life Nijmegen Cochlear Implantation Questionnaire (NCIQ) and its subdomains over the period of 2 years following cochlear implantation (CI)**. Shown are mean values and the 95% CI. Pre CI, before CI; post 6, post 12, and post 24, 6, 12, and 24 months after surgery. 1 basic speech perception; 2 advanced speech perception; 3 speech production; 4 self-esteem; 5 activity; and 6 social interactions.

The HRQoL measured by NCIQ also improved significantly, and the improvement was sustained over the period of study (Figure 4). In detail, the scales measuring basic sound perception, advanced sound perception, self-esteem, activity, and social interactions improved significantly 6 months after CI and remained so over the 24 months of the follow-up period. The only scale without statistically significant changes but with a trend toward improvement was “speech production” (Table 3).

**Figure 5:**
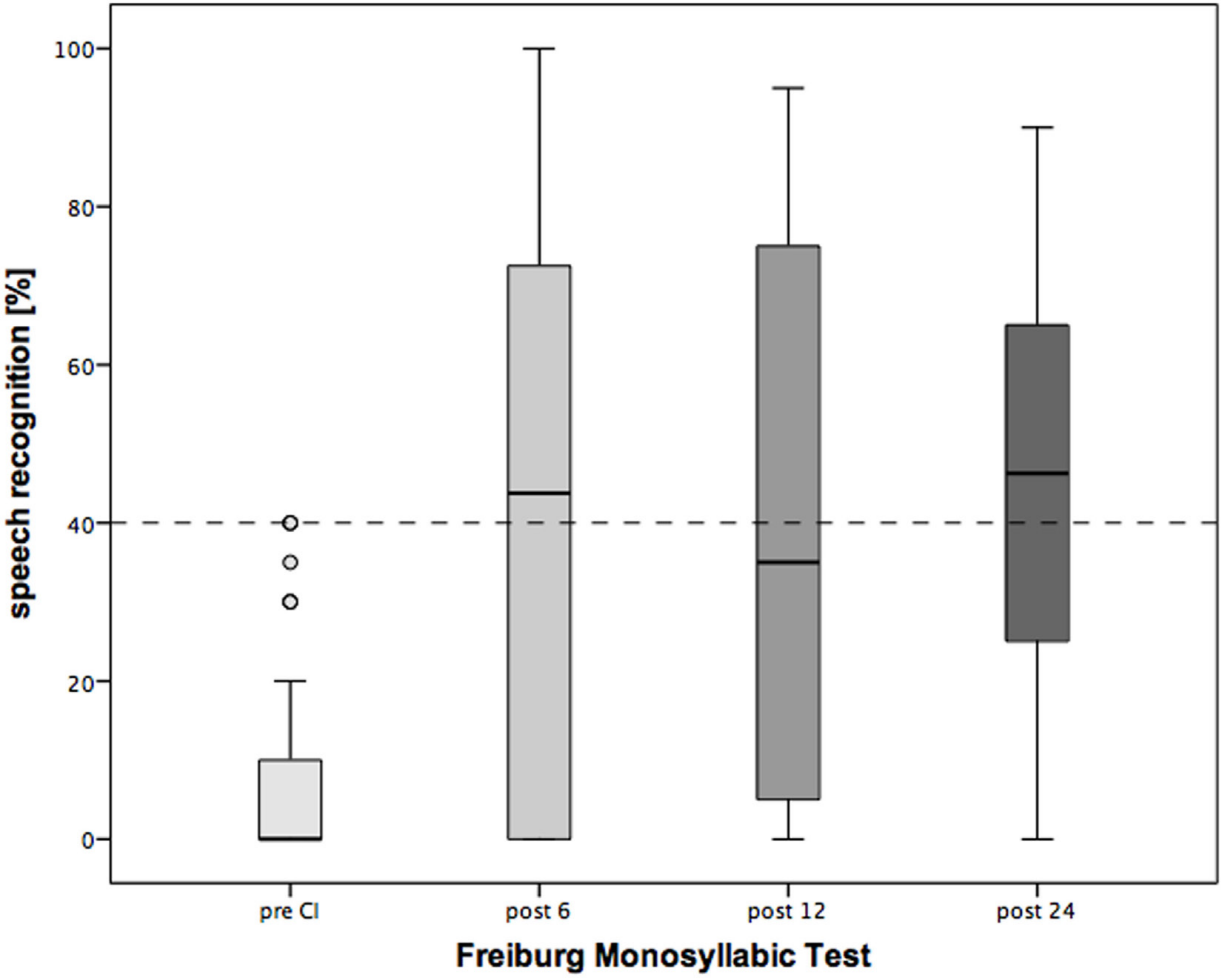
**Changes in speech recognition over the period of 2 years following cochlear implantation (CI)**. Shown are mean values and the range. Shown are mean values and the 95% CI. Dotted line: clinical criteria for CI.

Six months after CI, Oldenburg Inventory (Figure 5) demonstrated significant improvement in speech understanding in quiet and noise, as well speech localization at all measured time points of the follow-up period (Table 3). Similarly, monosyllabic Freiburg test indicated significant recovery of the hearing abilities by the implanted ear (Table 3). The speech recognition improved rapidly after surgery and was stable during the observation period of 2 years.

### Relationship between TQ and NCIQ

To determine if and how tinnitus-related distress affects the HRQoL, we computed the Spearman correlation coefficient for the respective variables. First, we analyzed the data obtained before CI (Table 4). We observed negative correlation between total TQ score and speech production (NCIQ3). The subscales indicating cognitive and emotional subscales as well as auditory difficulties reported by TQ were particularly affected. In addition, somatic complains correlated negatively with the background—and advanced sound perception as well as with self-esteem and social interactions (Table 4). Six months after CI, we found significant negative correlations between the total TQ score and all subdomains of NCIQ (Table 5), and 12 months after the CI, this was also the case (Table 6). Interestingly, 24 months after the CI, the correlations between total TQ score and NCIQ subscales “self-esteem” and “social interaction” were no longer significant (Table 7).

**Table 4:** **Correlation between tinnitus and health-related quality of life *before* cochlear implantation**.

			NCIQ1	NCIQ2	NCIQ3	NCIQ4	NCIQ5	NCIQ6	NCIQ total
Spearman-Rho	TQ E	Correlation coefficient	−0.202	−0.093	−0.313*	−0.097	−0.174	−0.146	−0.162
		Significance	0.205	0.561	0.046	0.548	0.276	0.362	0.313
		*N*	41	41	41	41	41	41	41
	TQ C	Correlation coefficient *t*	−0.118	−0.113	−0.315*	−0.144	−0.257	−0.240	−0.187
		Significance	0.464	0.483	0.045	0.370	0.105	0.131	0.241
		*N*	41	41	41	41	41	41	41
	TQ E + C	Correlation coefficient	−0.172	−0.108	−0.324*	−0.130	−0.232	−0.204	−0.191
		Significance	0.281	0.500	0.039	0.417	0.145	0.202	0.233
		*N*	41	41	41	41	41	41	41
	TQ I	Correlation coefficient	−0.287	−0.074	−0.300	−0.104	−0.210	−0.159	−0.199
		Sig. (2-seitig)	0.069	0.647	0.057	0.516	0.187	0.322	0.212
		*N*	41	41	41	41	41	41	41
	TQ A	Correlation coefficient	−0.258	−0.210	−0.437**	0.031	−0.044	0.043	−0.142
		Significance	0.104	0.187	0.004	0.849	0.784	0.791	0.378
		*N*	41	41	41	41	41	41	41
	TQ SI	Correlation coefficient	−0.354*	−0.315*	−0.263	−0.124	−0.279	−0.140	−0.259
		Significance	0.023	0.045	0.097	0.440	0.077	0.384	0.102
		*N*	41	41	41	41	41	41	41
	TQ SO	Correlation coefficient	−0.408**	−0.407**	−0.253	−0.274	−0.432**	−0.278	−0.413**
		Significance	0.008	0.008	0.111	0.083	0.005	0.078	0.007
		*N*	41	41	41	41	41	41	41
	TQ total	Correlation coefficient	−0.299	−0.205	−0.405**	−0.126	−0.243	−0.173	−0.260
		Significance	0.057	0.199	0.009	0.434	0.126	0.280	0.101
		*N*	41	41	41	41	41	41	41

*p ≤ 0.05.

**p ≤ 0.01.

*Subscales of Nijmegen Cochlear Implantation Questionnaire (NCIQ): 1 basic sound perception; 2 advanced sound perception; 3 speech production; 4 self-esteem; 5 activity; and 6 social interactions*.

*Subscales of Tinnitus Questionnaire (TQ): E, emotional distress; C, cognitive distress; E + C, combined psychological distress; I, intrusiveness; A, auditory perception difficulties; SI, sleep disturbances; SO, somatic complaints*.

**Table 5:** **Correlation between tinnitus and health-related quality of life *6 months after* cochlear implantation**.

			NCIQ1	NCIQ2	NCIQ3	NCIQ4	NCIQ5	NCIQ6	NCIQ total
Spearman-Rho	TQ E	Correlation coefficient	−0.308	−0.397*	−0.421**	−0.331*	−0.553**	−0.365*	−0.462**
		Significance	0.050	0.010	0.006	0.034	0.000	0.019	0.002
		*N*	41	41	41	41	41	41	41
	TQ C	Correlation coefficient *t*	−0.347*	−0.399**	−0.446**	−0.421**	−0.586**	−0.413**	−0.524**
		Significance	0.026	0.010	0.003	0.006	0.000	0.007	0.000
		*N*	41	41	41	41	41	41	41
	TQ E + C	Correlation coefficient	−0.335*	−0.417**	−0.461**	−0.384*	−0.581**	−0.394*	−0.502**
		Significance	0.032	0.007	0.002	0.013	0.000	0.011	0.001
		*N*	41	41	41	41	41	41	41
	TQ I	Correlation coefficient	−0.378*	−0.466**	−0.486**	−0.368*	−0.613**	−0.444**	−0.535**
		Sig. (2-seitig)	0.015	0.002	0.001	0.018	0.000	0.004	0.000
		*N*	41	41	41	41	41	41	41
	TQ A	Correlation coefficient	−0.320*	−0.476**	−0.396*	−0.327*	−0.510**	−0.425**	−0.449**
		Significance	0.041	0.002	0.010	0.037	0.001	0.006	0.003
		*N*	41	41	41	41	41	41	41
	TQ SI	Correlation coefficient	−0.145	−0.185	−0.191	−0.357*	−0.527**	−0.240	−0.344*
		Significance	0.365	0.246	0.231	0.022	0.000	0.131	0.028
		*N*	41	41	41	41	41	41	41
	TQ SO	Correlation coefficient	−0.381*	−0.398**	−0.390*	−0.294	−0.457**	−0.413**	−0.449**
		Significance	0.014	0.010	0.012	0.062	0.003	0.007	0.003
		*N*	41	41	41	41	41	41	41
	TQ Total	Correlation coefficient	−0.331*	−0.435**	−0.430**	−0.359*	−0.589**	−0.404**	−0.497**
		Significance	0.035	0.004	0.005	0.021	0.000	0.009	0.001
		*N*	41	41	41	41	41	41	41

*p ≤ 0.05.

**p ≤ 0.01.

*Subscales of Nijmegen Cochlear Implantation Questionnaire (NCIQ): 1 basic sound perception; 2 advanced sound perception; 3 speech production; 4 self-esteem; 5 activity; and 6 social interactions*.

*Subscales of Tinnitus Questionnaire (TQ): E, emotional distress; C, cognitive distress; E + C, combined psychological distress; I, intrusiveness; A, auditory perception difficulties; SI, sleep disturbances; SO, somatic complaints*.

**Table 6:** **Correlation between tinnitus and health-related quality of life *12 months after* cochlear implantation**.

			NCIQ1	NCIQ2	NCIQ3	NCIQ4	NCIQ5	NCIQ6	NCIQ total
Spearman-Rho	TQ E	Correlation coefficient	−0.377*	−0.545**	−0.362*	−0.329*	−0.409**	−0.386*	−0.400*
		Significance	0.018	0.000	0.024	0.041	0.010	0.015	0.010
		*N*	39	39	39	39	39	39	40
	TQ C	Correlation coefficient *t*	−0.505**	−0.593**	−0.372*	−0.408**	−0.477**	−0.447**	−0.488**
		Significance	0.001	0.000	0.020	0.010	0.002	0.004	0.001
		*N*	39	39	39	39	39	39	40
	TQ E + C	Correlation coefficient	−0.424**	−0.558**	−0.368*	−0.340*	−0.414**	−0.402*	−0.424**
		Significance	0.007	0.000	0.021	0.034	0.009	0.011	0.006
		*N*	39	39	39	39	39	39	40
	TQ I	Correlation coefficient	−0.454**	−0.598**	−0.490**	−0.332*	−0.472**	−0.446**	−0.490**
		Sig. (2-seitig)	0.004	0.000	0.002	0.039	0.002	0.004	0.001
		*N*	39	39	39	39	39	39	40
	TQ A	Correlation coefficient	−0.345*	−0.456**	−0.414**	−0.308	−0.348*	−0.306	−0.385*
		Significance	0.032	0.004	0.009	0.056	0.030	0.058	0.014
		*N*	39	39	39	39	39	39	40
	TQ SI	Correlation coefficient	−0.339*	−0.401*	−0.332*	−0.254	−0.350*	−0.307	−0.314*
		Significance	0.035	0.011	0.039	0.119	0.029	0.057	0.048
		*N*	39	39	39	39	39	39	40
	TQ SO	Correlation coefficient	−0.309	−0.356*	−0.331*	−0.323*	−0.358*	−0.433**	−0.312*
		Significance	0.056	0.026	0.039	0.045	0.025	0.006	0.050
		*N*	39	39	39	39	39	39	40
	TQ total	Correlation coefficient	−0.404*	−0.531**	−0.408**	−0.323*	−0.411**	−0.403*	−0.417**
		Significance	0.011	0.001	0.010	0.045	0.009	0.011	0.007
		*N*	39	39	39	39	39	39	40

*p ≤ 0.05.

**p ≤ 0.01.

*Subscales of Nijmegen Cochlear Implantation Questionnaire (NCIQ): 1 basic sound perception; 2 advanced sound perception; 3 speech production; 4 self-esteem; 5 activity; and 6 social interactions*.

*Subscales of Tinnitus Questionnaire (TQ): E, emotional distress; C, cognitive distress; E + C, combined psychological distress; I, intrusiveness; A, auditory perception difficulties; SI, sleep disturbances; SO, somatic complaints*.

**Table 7:** **Correlation between tinnitus and health-related quality of life *24 months after* cochlear implantation**.

			NCIQ1	NCIQ2	NCIQ3	NCIQ4	NCIQ5	NCIQ6	NCIQ total
Spearman-Rho	TQ E	Correlation coefficient	−0.416**	−0.499**	−0.358*	−0.174	−0.384*	−0.346*	−0.328*
		Significance	0.008	0.001	0.023	0.282	0.014	0.029	0.039
		*N*	40	40	40	40	40	40	40
	TQ C	Correlation coefficient *t*	−0.408**	−0.452**	−0.359*	−0.170	−0.341*	−0.277	−0.353*
		Significance	0.009	0.003	0.023	0.296	0.031	0.084	0.026
		*N*	40	40	40	40	40	40	40
	TQ E + C	Correlation coefficient	−0.406**	−0.474**	−0.362*	−0.165	−0.351*	−0.308	−0.327*
		Significance	0.009	0.002	0.022	0.308	0.026	0.053	0.039
		*N*	40	40	40	40	40	40	40
	TQ I	Correlation coefficient	−0.434**	−0.496**	−0.357*	−0.215	−0.411**	−0.351*	−0.339*
		Sig. (2-seitig)	0.005	0.001	0.024	0.183	0.008	0.026	0.032
		*N*	40	40	40	40	40	40	40
	TQ A	Correlation coefficient	−0.301	−0.462**	−0.439**	−0.135	−0.393*	−0.280	−0.257
		Significance	0.060	0.003	0.005	0.405	0.012	0.080	0.109
		*N*	40	40	40	40	40	40	40
	TQ SI	Correlation coefficient	−0.415**	−0.434**	−0.522**	−0.176	−0.385*	−0.277	−0.396*
		Significance	0.008	0.005	0.001	0.278	0.014	0.083	0.011
		*N*	40	40	40	40	40	40	40
	TQ SO	Correlation coefficient	−0.413**	−0.460**	−0.357*	−0.210	−0.409**	−0.355*	−0.371*
		Significance	0.008	0.003	0.024	0.194	0.009	0.025	0.019
		*N*	40	40	40	40	40	40	40
	TQ total	Correlation coefficient	−0.419**	−0.526**	−0.416**	−0.169	−0.372*	−0.311	−0.334*
		Significance	0.007	0.000	0.008	0.297	0.018	0.051	0.035
		*N*	40	40	40	40	40	40	40

*p ≤ 0.05.

**p ≤ 0.01.

*Subscales of Nijmegen Cochlear Implantation Questionnaire (NCIQ): 1 basic sound perception; 2 advanced sound perception; 3 speech production; 4 self-esteem; 5 activity; and 6 social interactions*.

*Subscales of Tinnitus Questionnaire (TQ): E, emotional distress; C, cognitive distress; E + C, combined psychological distress; I, intrusiveness; A, auditory perception difficulties; SI, sleep disturbances; SO, somatic complaints*.

### Compliance

Of 42 subjects originally included in this study, 41 patients filled the NCIQ questionnaire at the study onset, 39 patients after 12 months, and 40 patients after 2 years.

## Discussion

Tinnitus is often a symptom of hearing loss. Here, we demonstrated that in the bilaterally hearing-impaired patients with tinnitus, CI not only restores the auditory abilities but also reduces tinnitus-related distress and that this reduction was sustained for 2 years following surgery. To the best of our knowledge, our present study demonstrates for the first time the course of tinnitus-related distress in a homogenous cohort of bilaterally hard of hearing and tinnitus-positive patients, before and after CI. In addition, we show the relationship between tinnitus-related distress and the HRQoL and the postoperative auditory improvement over the 2-year course.

Prior to CI, the TQ score (total and subscales “emotional and cognitive distress” and “auditory difficulties”) correlated significantly with the third subscale of the HRQoL NCIQ “speech production,” whereas the total score of NCIQ correlated significantly (negative correlation) with the TQ subscale “somatic complaints” (Table 4). All correlations between NCIQ and TQ were negative, meaning that the decrease of tinnitus-related distress correlated with improvement of the quality of life and *vice versa*. Although these correlations decreased with time, they remained significant throughout the 24 months of the follow-up period (Tables 5–7), suggesting that the tinnitus-related emotional and cognitive distress as well as tinnitus-related auditory difficulties negatively influenced the life quality of the CI patients. Longer follow-up time should clarify if these correlations decay completely with time.

Before the CI, patients’ quality of life (total score) was not affected by the tinnitus-induced auditory difficulties (Table 4) confirming our earlier observations (13). Six months after implantation, there was a large (Rho = −0.449) and significant (*p* = 0.003) negative correlation between these two variables (Table 5), very likely reflecting the fact that the process of regaining auditory abilities can be negatively affected by the tinnitus percept. In fact, this correlation and its significance declined 12 months after CI (Rho = −0.385, *p* = 0.014) (Table 6) and were no longer of significance 24 months after the surgery (Table 7).

Tinnitus is a complaint of 70–90% of hearing-impaired patients (12–14). In cases of patients who are bilaterally hard of hearing, tinnitus percept is a particularly disturbing symptom, because it is the only auditory input perceived by patients. In such cases, diverting the auditory attention from tinnitus to other sounds is problematic, making the therapeutic approaches difficult if not impossible. The two major therapies globally used for tinnitus treatment are tinnitus retraining therapy (TRT) and cognitive behavioral therapy (CBT). The neurophysiological model proposed by Jastreboff (27, 28) suggests the existence of auditory–limbic–sympathetic network responsible for negative effects of tinnitus sound and inducing the distress and inability to divert the attention of patients from the tinnitus sound. TRT, designed by Jastreboff based on the above theory, has since years been a frequent therapeutic choice of many clinicians (29–31). The second method widely used for tinnitus is the CBT, which was developed to treat anxiety disorders, depression, eating disorders, chronic low back pain, personality disorders, depression, and anxiety and successfully applied in the treatment for tinnitus (32–35). TRT, CBT, or a combination of both require at least some hearing abilities and can only be used to treat the patients who are hard of hearing *and* have tinnitus following successful auditory rehabilitation with CI.

The first positive effect of CI on tinnitus was reported in 1976 by House (15). Ever since, various studies with different sample sizes and inclusion criteria were performed. Corroborating our present results, the decrease of tinnitus-related distress after CI ranging from 46 to 95% was observed previously by others (12, 36, 37). In our present study, we also observed the reduction of tinnitus-related distress in half of the patients who had severe (decompensated) tinnitus prior to CI.

The central question addressing the mechanism in which CI reduces tinnitus-related distress remains open. The evidence collected in our present study suggests following possible scenarios:

- Following CI, the auditory abilities improve to the degree where the patients can focus their auditory attention on sounds other than tinnitus.
- Following CI, the improved auditory abilities increase the quality of life, thus decrease overall stress and positively affect the loop “stress-tinnitus.”
- Following CI, the direct electrical stimulation of the auditory nerve induces plastic changes in the auditory reducing the tinnitus percept.

More quality evidence needs to be accumulated to determine, which of the presented scenarios is essential for tinnitus reduction after CI. It can also not be excluded that all three mechanisms play a role in tinnitus reduction and habituation. Future trials with specific tinnitus-oriented fitting of cochlear implants could shed more light on that topic.

While until recently, the clinical research involving cochlear implants was focused mainly on the audiological gain; at present, many authors are increasingly interested in changes of the quality of life and tinnitus-related distress (12, 38, 39). Quaranta et al. reported bilateral disappearance of tinnitus after unilateral CI in 65.8% of the patients (40), as measured by the Tinnitus Handicap Inventory—an instrument that is similar—but not identical—to TQ (41). Also, we have demonstrated earlier that the CI, in addition to having positive effect on hearing abilities, improves the life quality and decreases the tinnitus-related distress and psychological comorbidities (13, 42–45). There are several psychometric instruments measuring various parameters and domains used in tinnitus research and clinical routine. These instruments vary depending on the clinical orientation of the treating unit (audiology, ORL, clinical psychology or psychosomatic medicine) and on the country. Here, we propose creation of a specific set of standardized, validated, and internationally available instruments to measure CI-specific outcomes, which would include various aspects of tinnitus percept and tinnitus-related distress. In our present work, we used instruments that are widely available in the German-speaking countries. The Nijmegen Cochlear Implant Questionnaire NCIQ is an internationally validated, disease-specific instrument created for the assessment of the quality of life in patients with cochlear implants. OI is a popular, standardized instrument measuring perceived benefit of hearing aids. Also, the German version of TQ, which measures the tinnitus-related distress, is frequently used in the inpatient and outpatient settings to monitor the severity of tinnitus and its response to treatment. In order to study the influence of tinnitus on the outcome of CI, we suggest designing prospective, longitudinal clinical trials and using defined monitoring batch. Despite using such design, our present study is not free of pitfalls, as it could have included larger sample, and it was neither double blinded nor randomized. In addition, an appropriate control group is lacking. However, blinded and randomized design in the field of cochlear implant is difficult to be implemented because of specific features of the CI treatment, preventing the design of high-level evidence studies (19). Control group, which for instance could comprise patients who were implanted but their cochlear implants remain switched off, cannot be used because of obvious ethical reasons.

Previously reported high prevalence of tinnitus in the hearing-impaired patients puts the choice of tinnitus treatment in these patients up for discussion (12). In particular, the task of developing appropriate approach for the tinnitus treatment in bilaterally hearing-impaired patients remains open. The increasing incidence of hearing impairments, including the age-related hearing loss in context of demographic changes in our society, emphasizes the need for improvement in the therapy guidelines (46).

In addition, although we observed the most pronounced decrease of tinnitus-related distress 12 months after the implantation, the maximal correlation between TQ score and speech recognition (Table 3) occurred 6 months after implantation. These results do not contradict each other; rather, they point at the dependence of auditory rehabilitation on tinnitus treatment. Since the auditory benefit is patient specific, it is difficult to measure. Speech recognition—a typical parameter that measures hearing improvement—when used alone is not enough to act as an adequate indicator of tinnitus suppression. This is reflected by the results obtained 2 years after CI. Similarly, the TQ scores suggest that a unilateral acoustic stimulation with noticeable postoperative asymmetry does not lead to an unfavorable influence on the tinnitus-related distress, even over several years. The bilateral CI could be an ultimate target of hearing rehabilitation. In fact, sustained improvement of TQ scores was observed in 40 patients subjected to sequential bilateral CI (25).

### Final Conclusion

Taken together, our results suggest that patients who are bilaterally hard of hearing and have tinnitus profit from CI not only by regaining their auditory skills but also by a significant and sustained improvement of the HRQoL and reduction of tinnitus-related distress. Moreover, the negative correlation between tinnitus and the HRQoL indicates the importance of tinnitus as an obstacle in auditory rehabilitation of CI patients. It is tempting to speculate that therapy for tinnitus used after CI would further decrease tinnitus-related distress and, therefore, could increase the quality of life in this specific group of patients.

## Ethics Statement

The local Ethics Committee (permit number EA2/030/13) approved this prospective, non-interventional, and longitudinal study. All investigations were conducted according to the principles expressed in the Declaration of Helsinki. All patients have given their informed written consent.

## Author Contributions

SK and HO designed the study. SK, SG, and SH collected the data. SK, AS, and HO interpreted the data. SK and AS drafted the manuscript. HO critically revised the manuscript.

## Conflict of Interest Statement

The authors declare that the research was conducted in the absence of any commercial or financial relationships that could be construed as a potential conflict of interest.

## References

[1] Axelsson A Sandh A. Tinnitus in noise-induced hearing loss. Br J Audiol (1985) 19:271–6. 10.3109/03005368509078983 4074979

[2] Griest SE Bishop PM. Tinnitus as an early indicator of permanent hearing loss. A 15 year longitudinal study of noise exposed workers. AAOHN J (1998) 46:325–9. 9748912

[3] Mazurek B Olze H Haupt H Szczepek AJ. The more the worse: the grade of noise-induced hearing loss associates with the severity of tinnitus. Int J Environ Res Public Health (2010) 7:3071–9. 10.3390/ijerph7083071 20948948 PMC2954569

[4] Saltzman M Ersner MS A hearing aid for the relief of tinnitus aurium. Laryngoscope (1947) 57:358–66. 10.1288/00005537-194705000-00005 20241853

[5] Hoare DJ Edmondson-Jones M Sereda M Akeroyd MA Hall D Amplification with hearing aids for patients with tinnitus and co-existing hearing loss. Cochrane Database Syst Rev (2014) (1):CD010151 10.1002/14651858.CD010151.pub2 24482186 PMC10559339

[6] Fujimoto C Ito K Takano S Karino S Iwasaki S. Successful cochlear implantation in a patient with bilateral progressive sensorineural hearing loss after traumatic subarachnoid hemorrhage and brain contusion. Ann Otol Rhinol Laryngol (2007) 116:897–901. 10.1177/000348940711601205 18217508

[7] Suzuki Y Ogawa H Baba Y Suzuki T Yamada N Omori K. Cochlear implantation in a case of bilateral sensorineural hearing loss due to mumps. Fukushima J Med Sci (2009) 55:32–8. 10.5387/fms.55.32 19999167

[8] Greenberg SL Shipp D Lin VY Chen JM Nedzelski JM. Cochlear implantation in patients with bilateral severe sensorineural hearing loss after major blunt head trauma. Otol Neurotol (2011) 32:48–54. 10.1097/MAO.0b013e3181ff73fd 21157292

[9] Aoki M Tanahashi S Mizuta K Kato H. Treatment for progressive hearing loss due to Paget’s disease of bone – a case report and literature review. J Int Adv Otol (2015) 11:267–70. 10.5152/iao.2015.1572 26915163

[10] Ramakers GG Van Zon A Stegeman I Grolman W. The effect of cochlear implantation on tinnitus in patients with bilateral hearing loss: a systematic review. Laryngoscope (2015) 125:2584–92. 10.1002/lary.25370 26153087

[11] Lin FR Niparko JK Ferrucci L Hearing loss prevalence in the United States. Arch Intern Med (2011) 171:1851–2. 10.1001/archinternmed.2011.506 22083573 PMC3564588

[12] Baguley DM Atlas MD Cochlear implants and tinnitus. Prog Brain Res (2007) 166:347–55. 10.1016/S0079-6123(07)66033-6 17956799

[13] Olze H Szczepek AJ Haupt H Forster U Zirke N Grabel S Cochlear implantation has a positive influence on quality of life, tinnitus, and psychological comorbidity. Laryngoscope (2011) 121:2220–7. 10.1002/lary.22145 21898434

[14] Olze H Szczepek AJ Haupt H Zirke N Graebel S Mazurek B The impact of cochlear implantation on tinnitus, stress and quality of life in postlingually deafened patients. Audiol Neurootol (2012) 17:2–11. 10.1159/000323847 21540584

[15] House WF Cochlear implants. Ann Otol Rhinol Laryngol (1976) 85:3–93. 10.1177/00034894760850S303 779582

[16] Thedinger B House WF Edgerton BJ. Cochlear implant for tinnitus. Case reports. Ann Otol Rhinol Laryngol (1985) 94:10–3. 10.1177/000348948509400102 3838225

[17] House D Tinnitus suppression via cochlear implants: review and remarks. Int Tinnitus J (1999) 5:27–9. 10753414

[18] Arts RA George EL Stokroos RJ Vermeire K. Review: cochlear implants as a treatment of tinnitus in single-sided deafness. Curr Opin Otolaryngol Head Neck Surg (2012) 20:398–403. 10.1097/MOO.0b013e3283577b66 22931903

[19] Van Zon A Peters JP Stegeman I Smit AL Grolman W. Cochlear implantation for patients with single-sided deafness or asymmetrical hearing loss: a systematic review of the evidence. Otol Neurotol (2015) 36:209–19. 10.1097/MAO.0000000000000681 25502451

[20] Hall DA Haider H Szczepek AJ Lau P Rabau S Jones-Diette J Systematic review of outcome domains and instruments used in clinical trials of tinnitus treatments in adults. Trials (2016) 17:270. 10.1186/s13063-016-1399-9 27250987 PMC4888312

[21] Gordon KA Jiwani S Papsin BC. Benefits and detriments of unilateral cochlear implant use on bilateral auditory development in children who are deaf. Front Psychol (2013) 4:719. 10.3389/fpsyg.2013.00719 24137143 PMC3797443

[22] Gartrell BC Jones HG Kan A Buhr-Lawler M Gubbels SP Litovsky RY. Investigating long-term effects of cochlear implantation in single-sided deafness: a best practice model for longitudinal assessment of spatial hearing abilities and tinnitus handicap. Otol Neurotol (2014) 35:1525–32. 10.1097/MAO.0000000000000437 25158615 PMC4334463

[23] Ramos Macias A Falcon Gonzalez JC Manrique M Morera C Garcia-Ibanez L Cenjor C Cochlear implants as a treatment option for unilateral hearing loss, severe tinnitus and hyperacusis. Audiol Neurootol (2015) 20(Suppl 1):60–6. 10.1159/000380750 25997672

[24] Hirschfelder A Grabel S Olze H. The impact of cochlear implantation on quality of life: the role of audiologic performance and variables. Otolaryngol Head Neck Surg (2008) 138:357–62. 10.1016/j.otohns.2007.10.019 18312885

[25] Olze H Grabel S Haupt H Forster U Mazurek B Extra benefit of a second cochlear implant with respect to health-related quality of life and tinnitus. Otol Neurotol (2012) 33:1169–75. 10.1097/MAO.0b013e31825e799f 22892805

[26] Goebel G Hiller W. [The Tinnitus Questionnaire. A standard instrument for grading the degree of tinnitus. Results of a multicenter study with the Tinnitus Questionnaire]. HNO (1994) 42:166–72. 8175381

[27] Jastreboff PJ. Phantom auditory perception (tinnitus): mechanisms of generation and perception. Neurosci Res (1990) 8:221–54. 10.1016/0168-0102(90)90031-9 2175858

[28] Jastreboff PJ Hazell JW Graham RL. Neurophysiological model of tinnitus: dependence of the minimal masking level on treatment outcome. Hear Res (1994) 80:216–32. 10.1016/0378-5955(94)90113-9 7896580

[29] Kroener-Herwig B Biesinger E Gerhards F Goebel G Verena Greimel K Hiller W. Retraining therapy for chronic tinnitus. A critical analysis of its status. Scand Audiol (2000) 29:67–78. 10.1080/010503900424471 10888343

[30] Bartnik G Fabijańska A Rogowski M. Effects of tinnitus retraining therapy (TRT) for patients with tinnitus and subjective hearing loss versus tinnitus only. Scand Audiol (2001) 30(Suppl 52):206–8. 10.1080/010503901300007542 11318470

[31] Seydel C Haupt H Szczepek AJ Hartmann A Rose M Mazurek B. Three years later: report on the state of well-being of patients with chronic tinnitus who underwent modified tinnitus retraining therapy. Audiol Neurootol (2015) 20:26–38. 10.1159/000363728 25413891

[32] Andersson G. Psychological aspects of tinnitus and the application of cognitive-behavioral therapy. Clin Psychol Rev (2002) 22:977–90. 10.1016/S0272-7358(01)00124-6 12238249

[33] Hesser H Weise C Westin VZ Andersson G. A systematic review and meta-analysis of randomized controlled trials of cognitive-behavioral therapy for tinnitus distress. Clin Psychol Rev (2011) 31:545–53. 10.1016/j.cpr.2010.12.006 21237544

[34] Jun HJ Park MK. Cognitive behavioral therapy for tinnitus: evidence and efficacy. Korean J Audiol (2013) 17:101–4. 10.7874/kja.2013.17.3.101 24653916 PMC3936550

[35] Zenner HP Vonthein R Zenner B Leuchtweis R Plontke SK Torka W Standardized tinnitus-specific individual cognitive-behavioral therapy: a controlled outcome study with 286 tinnitus patients. Hear Res (2013) 298:117–25. 10.1016/j.heares.2012.11.013 23287811

[36] Quaranta N Wagstaff S Baguley DM. Tinnitus and cochlear implantation. Int J Audiol (2004) 43:245–51. 10.1080/14992020400050033 15357407

[37] Pan T Tyler RS Ji H Coelho C Gehringer AK Gogel SA. Changes in the tinnitus handicap questionnaire after cochlear implantation. Am J Audiol (2009) 18:144–51. 10.1044/1059-0889(2009/07-0042) 19949236 PMC2952398

[38] Andersson G Freijd A Baguley DM Idrizbegovic E. Tinnitus distress, anxiety, depression, and hearing problems among cochlear implant patients with tinnitus. J Am Acad Audiol (2009) 20:315–9. 10.3766/jaaa.20.5.5 19585962

[39] Contrera KJ Betz J Li L Blake CR Sung YK Choi JS Quality of life after intervention with a cochlear implant or hearing aid. Laryngoscope (2016) 126:2110–5. 10.1002/lary.25848 26775283 PMC4947575

[40] Quaranta N Fernandez-Vega S D’elia C Filipo R Quaranta A. The effect of unilateral multichannel cochlear implant on bilaterally perceived tinnitus. Acta Otolaryngol (2008) 128:159–63. 10.1080/00016480701387173 17851950

[41] Zeman F Koller M Schecklmann M Langguth B Landgrebe M TRI Database Study Group. Tinnitus assessment by means of standardized self-report questionnaires: psychometric properties of the Tinnitus Questionnaire (TQ), the Tinnitus Handicap Inventory (THI), and their short versions in an international and multi-lingual sample. Health Qual Life Outcomes (2012) 10:128. 10.1186/1477-7525-10-128 23078754 PMC3541124

[42] Olze H Grabel S Forster U Zirke N Huhnd LE Haupt H Elderly patients benefit from cochlear implantation regarding auditory rehabilitation, quality of life, tinnitus, and stress. Laryngoscope (2012) 122:196–203. 10.1002/lary.22356 21997855

[43] Bruggemann P Szczepek AJ Rose M Mckenna L Olze H Mazurek B. Impact of multiple factors on the degree of tinnitus distress. Front Hum Neurosci (2016) 10:341. 10.3389/fnhum.2016.00341 27445776 PMC4925660

[44] Knopke S Grabel S Forster-Ruhrmann U Mazurek B Szczepek AJ Olze H. Impact of cochlear implantation on quality of life and mental comorbidity in patients aged 80 years. Laryngoscope (2016) 126:2811–6. 10.1002/lary.25993 27075602

[45] Olze H Knopke S Grabel S Szczepek AJ. Rapid positive influence of cochlear implantation on the quality of life in adults 70 years and older. Audiol Neurootol (2016) 21(Suppl 1):43–7. 10.1159/000448354 27806365

[46] Davis A Mcmahon CM Pichora-Fuller KM Russ S Lin F Olusanya BO Aging and hearing health: the life-course approach. Gerontologist (2016) 56(Suppl 2):S256–67. 10.1093/geront/gnw033 26994265 PMC6283365

